# Supplementary material for: Classification of Latilactobacillus sakei subspecies based on MALDI-TOF MS protein profiles using machine learning models
Source: Microbiol Spectr. 2024 Aug 20;12(10):e03668-23. doi: 10.1128/spectrum.03668-23 (PMC11448074; doi:10.1128/spectrum.03668-23)
Supplement: Supplemental material — Fig. S1; Table S1. [file spectrum.03668-23-s0001.docx]

**Supplementary Materials**

**
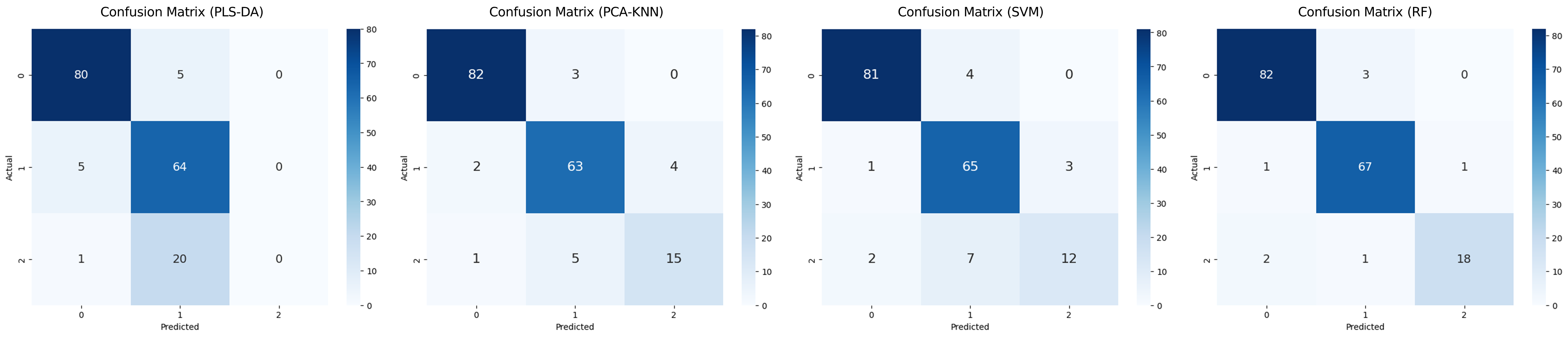
**

**Fig. S1.** Confusion matrix for classification performance of three machine learning models (PLS-DA, PCA-KNN, SVM, and RF). 0, 1, and 2 on actual and predicted labels indicate *L*. *sakei* subsp. *carnosus*, *L*. *sakei* subsp. *sakei*, and non-*L*. *sakei* species.

**Table S1.** List of strains isolated in this study

| Subspecies | Strain | Sample information | | |
| --- | --- | --- | --- | --- |
|  |  | Sample no. | Classification | Source |
| *L*. *sakei* subsp. *carnosus* | SC1 | Sample 1 | Fermented fish | Jogae-jeot |
| *L*. *sakei* subsp. *carnosus* | SC2 | Sample 1 | Fermented fish | Jogae-jeot |
| *L*. *sakei* subsp. *carnosus* | SC3 | Sample 1 | Fermented fish | Jogae-jeot |
| *L*. *sakei* subsp. *carnosus* | SC4 | Sample 1 | Fermented fish | Jogae-jeot |
| *L*. *sakei* subsp. *carnosus* | SC5 | Sample 1 | Fermented fish | Jogae-jeot |
| *L*. *sakei* subsp. *carnosus* | SC6 | Sample 1 | Fermented fish | Jogae-jeot |
| *L*. *sakei* subsp. *carnosus* | SC7 | Sample 2 | Fermented fish | Jogae-jeot |
| *L*. *sakei* subsp. *carnosus* | SC8 | Sample 2 | Fermented fish | Jogae-jeot |
| *L*. *sakei* subsp. *carnosus* | SC9 | Sample 2 | Fermented fish | Jogae-jeot |
| *L*. *sakei* subsp. *carnosus* | SC10 | Sample 2 | Fermented fish | Jogae-jeot |
| *L*. *sakei* subsp. *carnosus* | SC11 | Sample 2 | Fermented fish | Jogae-jeot |
| *L*. *sakei* subsp. *carnosus* | SC12 | Sample 2 | Fermented fish | Jogae-jeot |
| *L*. *sakei* subsp. *carnosus* | SC13 | Sample 2 | Fermented fish | Jogae-jeot |
| *L*. *sakei* subsp. *carnosus* | SC14 | Sample 2 | Fermented fish | Jogae-jeot |
| *L*. *sakei* subsp. *carnosus* | SC15 | Sample 2 | Fermented fish | Jogae-jeot |
| *L*. *sakei* subsp. *carnosus* | SC16 | Sample 3 | Fermented fish | Jogae-jeot |
| *L*. *sakei* subsp. *carnosus* | SC17 | Sample 3 | Fermented fish | Jogae-jeot |
| *L*. *sakei* subsp. *carnosus* | SC18 | Sample 3 | Fermented fish | Jogae-jeot |
| *L*. *sakei* subsp. *carnosus* | SC19 | Sample 3 | Fermented fish | Jogae-jeot |
| *L*. *sakei* subsp. *carnosus* | SC20 | Sample 3 | Fermented fish | Jogae-jeot |
| *L*. *sakei* subsp. *carnosus* | SC21 | Sample 3 | Fermented fish | Jogae-jeot |
| *L*. *sakei* subsp. *carnosus* | SC22 | Sample 3 | Fermented fish | Jogae-jeot |
| *L*. *sakei* subsp. *carnosus* | SC23 | Sample 3 | Fermented fish | Jogae-jeot |
| *L*. *sakei* subsp. *carnosus* | SC24 | Sample 3 | Fermented fish | Jogae-jeot |
| *L*. *sakei* subsp. *carnosus* | SC25 | Sample 3 | Fermented fish | Jogae-jeot |
| *L*. *sakei* subsp. *carnosus* | SC26 | Sample 4 | Fermented meat | Jamon |
| *L*. *sakei* subsp. *carnosus* | SC27 | Sample 4 | Fermented meat | Jamon |
| *L*. *sakei* subsp. *carnosus* | SC28 | Sample 4 | Fermented meat | Jamon |
| *L*. *sakei* subsp. *carnosus* | SC29 | Sample 4 | Fermented meat | Jamon |
| *L*. *sakei* subsp. *carnosus* | SC30 | Sample 4 | Fermented meat | Jamon |
| *L*. *sakei* subsp. *carnosus* | SC31 | Sample 4 | Fermented meat | Jamon |
| *L*. *sakei* subsp. *carnosus* | SC32 | Sample 4 | Fermented meat | Jamon |
| *L*. *sakei* subsp. *carnosus* | SC33 | Sample 4 | Fermented meat | Jamon |
| *L*. *sakei* subsp. *carnosus* | SC34 | Sample 5 | Fermented meat | Salami |
| *L*. *sakei* subsp. *carnosus* | SC35 | Sample 5 | Fermented meat | Salami |
| *L*. *sakei* subsp. *carnosus* | SC36 | Sample 5 | Fermented meat | Salami |
| *L*. *sakei* subsp. *carnosus* | SC37 | Sample 5 | Fermented meat | Salami |
| *L*. *sakei* subsp. *carnosus* | SC38 | Sample 5 | Fermented meat | Salami |
| *L*. *sakei* subsp. *carnosus* | SC39 | Sample 5 | Fermented meat | Salami |
| *L*. *sakei* subsp. *carnosus* | SC40 | Sample 5 | Fermented meat | Salami |
| *L*. *sakei* subsp. *carnosus* | SC41 | Sample 5 | Fermented meat | Salami |
| *L*. *sakei* subsp. *carnosus* | SC42 | Sample 6 | Fermented meat | Salami |
| *L*. *sakei* subsp. *carnosus* | SC43 | Sample 6 | Fermented meat | Salami |
| *L*. *sakei* subsp. *carnosus* | SC44 | Sample 6 | Fermented meat | Salami |
| *L*. *sakei* subsp. *carnosus* | SC45 | Sample 6 | Fermented meat | Salami |
| *L*. *sakei* subsp. *carnosus* | SC46 | Sample 6 | Fermented meat | Salami |
| *L*. *sakei* subsp. *carnosus* | SC47 | Sample 6 | Fermented meat | Salami |
| *L*. *sakei* subsp. *carnosus* | SC48 | Sample 6 | Fermented meat | Salami |
| *L*. *sakei* subsp. *carnosus* | SC49 | Sample 6 | Fermented meat | Salami |
| *L*. *sakei* subsp. *carnosus* | SC50 | Sample 6 | Fermented meat | Salami |
| *L*. *sakei* subsp. *carnosus* | SC51 | Sample 6 | Fermented meat | Salami |
| *L*. *sakei* subsp. *carnosus* | SC52 | Sample 6 | Fermented meat | Salami |
| *L*. *sakei* subsp. *carnosus* | SC53 | Sample 6 | Fermented meat | Salami |
| *L*. *sakei* subsp. *carnosus* | SC54 | Sample 6 | Fermented meat | Salami |
| *L*. *sakei* subsp. *carnosus* | SC55 | Sample 6 | Fermented meat | Salami |
| *L*. *sakei* subsp. *carnosus* | SC56 | Sample 7 | Fermented meat | Coppa |
| *L*. *sakei* subsp. *carnosus* | SC57 | Sample 7 | Fermented meat | Coppa |
| *L*. *sakei* subsp. *carnosus* | SC58 | Sample 7 | Fermented meat | Coppa |
| *L*. *sakei* subsp. *carnosus* | SC59 | Sample 7 | Fermented meat | Coppa |
| *L*. *sakei* subsp. *carnosus* | SC60 | Sample 7 | Fermented meat | Coppa |
| *L*. *sakei* subsp. *carnosus* | SC61 | Sample 7 | Fermented meat | Coppa |
| *L*. *sakei* subsp. *carnosus* | SC62 | Sample 8 | Fermented meat | Prosciutto |
| *L*. *sakei* subsp. *carnosus* | SC63 | Sample 8 | Fermented meat | Prosciutto |
| *L*. *sakei* subsp. *carnosus* | SC64 | Sample 8 | Fermented meat | Prosciutto |
| *L*. *sakei* subsp. *carnosus* | SC65 | Sample 8 | Fermented meat | Prosciutto |
| *L*. *sakei* subsp. *carnosus* | SC66 | Sample 8 | Fermented meat | Prosciutto |
| *L*. *sakei* subsp. *carnosus* | SC67 | Sample 9 | Fermented meat | Chorizo |
| *L*. *sakei* subsp. *carnosus* | SC68 | Sample 9 | Fermented meat | Chorizo |
| *L*. *sakei* subsp. *carnosus* | SC69 | Sample 9 | Fermented meat | Chorizo |
| *L*. *sakei* subsp. *carnosus* | SC70 | Sample 9 | Fermented meat | Chorizo |
| *L*. *sakei* subsp. *carnosus* | SC71 | Sample 9 | Fermented meat | Chorizo |
| *L*. *sakei* subsp. *carnosus* | SC72 | Sample 9 | Fermented meat | Chorizo |
| *L*. *sakei* subsp. *carnosus* | SC73 | Sample 9 | Fermented meat | Chorizo |
| *L*. *sakei* subsp. *carnosus* | SC74 | Sample 9 | Fermented meat | Chorizo |
| *L*. *sakei* subsp. *carnosus* | SC75 | Sample 9 | Fermented meat | Chorizo |
| *L*. *sakei* subsp. *carnosus* | SC76 | Sample 9 | Fermented meat | Chorizo |
| *L*. *sakei* subsp. *carnosus* | SC77 | Sample 10 | Fermented meat | Salami |
| *L*. *sakei* subsp. *carnosus* | SC78 | Sample 10 | Fermented meat | Salami |
| *L*. *sakei* subsp. *carnosus* | SC79 | Sample 10 | Fermented meat | Salami |
| *L*. *sakei* subsp. *carnosus* | SC80 | Sample 10 | Fermented meat | Salami |
| *L*. *sakei* subsp. *carnosus* | SC81 | Sample 10 | Fermented meat | Salami |
| *L*. *sakei* subsp. *carnosus* | SC82 | Sample 10 | Fermented meat | Salami |
| *L*. *sakei* subsp. *carnosus* | SC83 | Sample 10 | Fermented meat | Salami |
| *L*. *sakei* subsp. *carnosus* | SC84 | Sample 11 | Fermented meat | Salami |
| *L*. *sakei* subsp. *carnosus* | SC85 | Sample 11 | Fermented meat | Salami |
| *L*. *sakei* subsp. *carnosus* | SC86 | Sample 11 | Fermented meat | Salami |
| *L*. *sakei* subsp. *carnosus* | SC87 | Sample 11 | Fermented meat | Salami |
| *L*. *sakei* subsp. *carnosus* | SC88 | Sample 11 | Fermented meat | Salami |
| *L*. *sakei* subsp. *carnosus* | SC89 | Sample 11 | Fermented meat | Salami |
| *L*. *sakei* subsp. *carnosus* | SC90 | Sample 11 | Fermented meat | Salami |
| *L*. *sakei* subsp. *carnosus* | SC91 | Sample 11 | Fermented meat | Salami |
| *L*. *sakei* subsp. *carnosus* | SC92 | Sample 11 | Fermented meat | Salami |
| *L*. *sakei* subsp. *carnosus* | SC93 | Sample 12 | Fermented meat | Salami |
| *L*. *sakei* subsp. *carnosus* | SC94 | Sample 12 | Fermented meat | Salami |
| *L*. *sakei* subsp. *carnosus* | SC95 | Sample 12 | Fermented meat | Salami |
| *L*. *sakei* subsp. *carnosus* | SC96 | Sample 12 | Fermented meat | Salami |
| *L*. *sakei* subsp. *carnosus* | SC97 | Sample 12 | Fermented meat | Salami |
| *L*. *sakei* subsp. *carnosus* | SC98 | Sample 12 | Fermented meat | Salami |
| *L*. *sakei* subsp. *carnosus* | SC99 | Sample 12 | Fermented meat | Salami |
| *L*. *sakei* subsp. *carnosus* | SC100 | Sample 13 | Fermented meat | Salami |
| *L*. *sakei* subsp. *carnosus* | SC101 | Sample 13 | Fermented meat | Salami |
| *L*. *sakei* subsp. *carnosus* | SC102 | Sample 13 | Fermented meat | Salami |
| *L*. *sakei* subsp. *carnosus* | SC103 | Sample 14 | Fermented meat | Salchichon |
| *L*. *sakei* subsp. *carnosus* | SC104 | Sample 14 | Fermented meat | Salchichon |
| *L*. *sakei* subsp. *sakei* | SS1 | Sample 15 | Fermented fish | Jeonbok-jeot |
| *L*. *sakei* subsp. *sakei* | SS2 | Sample 15 | Fermented fish | Jeonbok-jeot |
| *L*. *sakei* subsp. *sakei* | SS3 | Sample 15 | Fermented fish | Jeonbok-jeot |
| *L*. *sakei* subsp. *sakei* | SS4 | Sample 15 | Fermented fish | Jeonbok-jeot |
| *L*. *sakei* subsp. *sakei* | SS5 | Sample 15 | Fermented fish | Jeonbok-jeot |
| *L*. *sakei* subsp. *sakei* | SS6 | Sample 15 | Fermented fish | Jeonbok-jeot |
| *L*. *sakei* subsp. *sakei* | SS7 | Sample 16 | Fermented fish | Jeonbok-jeot |
| *L*. *sakei* subsp. *sakei* | SS8 | Sample 16 | Fermented fish | Jeonbok-jeot |
| *L*. *sakei* subsp. *sakei* | SS9 | Sample 16 | Fermented fish | Jeonbok-jeot |
| *L*. *sakei* subsp. *sakei* | SS10 | Sample 16 | Fermented fish | Jeonbok-jeot |
| *L*. *sakei* subsp. *sakei* | SS11 | Sample 16 | Fermented fish | Jeonbok-jeot |
| *L*. *sakei* subsp. *sakei* | SS12 | Sample 16 | Fermented fish | Jeonbok-jeot |
| *L*. *sakei* subsp. *sakei* | SS13 | Sample 16 | Fermented fish | Jeonbok-jeot |
| *L*. *sakei* subsp. *sakei* | SS14 | Sample 17 | Fermented fish | Jeonbok-jeot |
| *L*. *sakei* subsp. *sakei* | SS15 | Sample 17 | Fermented fish | Jeonbok-jeot |
| *L*. *sakei* subsp. *sakei* | SS16 | Sample 17 | Fermented fish | Jeonbok-jeot |
| *L*. *sakei* subsp. *sakei* | SS17 | Sample 17 | Fermented fish | Jeonbok-jeot |
| *L*. *sakei* subsp. *sakei* | SS18 | Sample 17 | Fermented fish | Jeonbok-jeot |
| *L*. *sakei* subsp. *sakei* | SS19 | Sample 17 | Fermented fish | Jeonbok-jeot |
| *L*. *sakei* subsp. *sakei* | SS20 | Sample 17 | Fermented fish | Jeonbok-jeot |
| *L*. *sakei* subsp. *sakei* | SS21 | Sample 17 | Fermented fish | Jeonbok-jeot |
| *L*. *sakei* subsp. *sakei* | SS22 | Sample 17 | Fermented fish | Jeonbok-jeot |
| *L*. *sakei* subsp. *sakei* | SS41 | Sample 18 | Fermented fish | Abalone intestines |
| *L*. *sakei* subsp. *sakei* | SS42 | Sample 18 | Fermented fish | Abalone intestines |
| *L*. *sakei* subsp. *sakei* | SS43 | Sample 18 | Fermented fish | Abalone intestines |
| *L*. *sakei* subsp. *sakei* | SS44 | Sample 18 | Fermented fish | Abalone intestines |
| *L*. *sakei* subsp. *sakei* | SS45 | Sample 18 | Fermented fish | Abalone intestines |
| *L*. *sakei* subsp. *sakei* | SS46 | Sample 18 | Fermented fish | Abalone intestines |
| *L*. *sakei* subsp. *sakei* | SS47 | Sample 18 | Fermented fish | Abalone intestines |
| *L*. *sakei* subsp. *sakei* | SS37 | Sample 19 | Fermented fish | Myeolchi-jeot |
| *L*. *sakei* subsp. *sakei* | SS38 | Sample 19 | Fermented fish | Myeolchi-jeot |
| *L*. *sakei* subsp. *sakei* | SS39 | Sample 19 | Fermented fish | Myeolchi-jeot |
| *L*. *sakei* subsp. *sakei* | SS40 | Sample 19 | Fermented fish | Myeolchi-jeot |
| *L*. *sakei* subsp. *sakei* | SS55 | Sample 20 | Fermented fish | Myeolchi-jeot |
| *L*. *sakei* subsp. *sakei* | SS56 | Sample 20 | Fermented fish | Myeolchi-jeot |
| *L*. *sakei* subsp. *sakei* | SS48 | Sample 21 | Fermented fish | Jogae-jeot |
| *L*. *sakei* subsp. *sakei* | SS49 | Sample 21 | Fermented fish | Jogae-jeot |
| *L*. *sakei* subsp. *sakei* | SS50 | Sample 21 | Fermented fish | Jogae-jeot |
| *L*. *sakei* subsp. *sakei* | SS51 | Sample 21 | Fermented fish | Jogae-jeot |
| *L*. *sakei* subsp. *sakei* | SS52 | Sample 21 | Fermented fish | Jogae-jeot |
| *L*. *sakei* subsp. *sakei* | SS53 | Sample 21 | Fermented fish | Jogae-jeot |
| *L*. *sakei* subsp. *sakei* | SS54 | Sample 21 | Fermented fish | Jogae-jeot |
| *L*. *sakei* subsp. *sakei* | SS84 | Sample 22 | Fermented fish | Ojingeo-jeot |
| *L*. *sakei* subsp. *sakei* | SS85 | Sample 22 | Fermented fish | Ojingeo-jeot |
| *L*. *sakei* subsp. *sakei* | SS86 | Sample 22 | Fermented fish | Ojingeo-jeot |
| *L*. *sakei* subsp. *sakei* | SS23 | Sample 23 | Fermented vegetable | Kimchi |
| *L*. *sakei* subsp. *sakei* | SS24 | Sample 23 | Fermented vegetable | Kimchi |
| *L*. *sakei* subsp. *sakei* | SS25 | Sample 23 | Fermented vegetable | Kimchi |
| *L*. *sakei* subsp. *sakei* | SS26 | Sample 23 | Fermented vegetable | Kimchi |
| *L*. *sakei* subsp. *sakei* | SS27 | Sample 23 | Fermented vegetable | Kimchi |
| *L*. *sakei* subsp. *sakei* | SS28 | Sample 23 | Fermented vegetable | Kimchi |
| *L*. *sakei* subsp. *sakei* | SS29 | Sample 23 | Fermented vegetable | Kimchi |
| *L*. *sakei* subsp. *sakei* | SS30 | Sample 23 | Fermented vegetable | Kimchi |
| *L*. *sakei* subsp. *sakei* | SS31 | Sample 23 | Fermented vegetable | Kimchi |
| *L*. *sakei* subsp. *sakei* | SS32 | Sample 24 | Fermented vegetable | Kimchi |
| *L*. *sakei* subsp. *sakei* | SS33 | Sample 24 | Fermented vegetable | Kimchi |
| *L*. *sakei* subsp. *sakei* | SS34 | Sample 24 | Fermented vegetable | Kimchi |
| *L*. *sakei* subsp. *sakei* | SS35 | Sample 24 | Fermented vegetable | Kimchi |
| *L*. *sakei* subsp. *sakei* | SS36 | Sample 24 | Fermented vegetable | Kimchi |
| *L*. *sakei* subsp. *sakei* | SS57 | Sample 25 | Fermented vegetable | Kimchi |
| *L*. *sakei* subsp. *sakei* | SS58 | Sample 25 | Fermented vegetable | Kimchi |
| *L*. *sakei* subsp. *sakei* | SS68 | Sample 26 | Fermented vegetable | Kimchi |
| *L*. *sakei* subsp. *sakei* | SS69 | Sample 26 | Fermented vegetable | Kimchi |
| *L*. *sakei* subsp. *sakei* | SS70 | Sample 26 | Fermented vegetable | Kimchi |
| *L*. *sakei* subsp. *sakei* | SS71 | Sample 26 | Fermented vegetable | Kimchi |
| *L*. *sakei* subsp. *sakei* | SS72 | Sample 26 | Fermented vegetable | Kimchi |
| *L*. *sakei* subsp. *sakei* | SS91 | Sample 27 | Fermented vegetable | Kimchi |
| *L*. *sakei* subsp. *sakei* | SS59 | Sample 28 | Fermented vegetable | Water kimchi |
| *L*. *sakei* subsp. *sakei* | SS60 | Sample 28 | Fermented vegetable | Water kimchi |
| *L*. *sakei* subsp. *sakei* | SS61 | Sample 28 | Fermented vegetable | Water kimchi |
| *L*. *sakei* subsp. *sakei* | SS62 | Sample 28 | Fermented vegetable | Water kimchi |
| *L*. *sakei* subsp. *sakei* | SS63 | Sample 28 | Fermented vegetable | Water kimchi |
| *L*. *sakei* subsp. *sakei* | SS64 | Sample 28 | Fermented vegetable | Water kimchi |
| *L*. *sakei* subsp. *sakei* | SS65 | Sample 28 | Fermented vegetable | Water kimchi |
| *L*. *sakei* subsp. *sakei* | SS66 | Sample 28 | Fermented vegetable | Water kimchi |
| *L*. *sakei* subsp. *sakei* | SS67 | Sample 28 | Fermented vegetable | Water kimchi |
| *L*. *sakei* subsp. *sakei* | SS73 | Sample 29 | Fermented vegetable | Radish kimchi |
| *L*. *sakei* subsp. *sakei* | SS74 | Sample 29 | Fermented vegetable | Radish kimchi |
| *L*. *sakei* subsp. *sakei* | SS75 | Sample 29 | Fermented vegetable | Radish kimchi |
| *L*. *sakei* subsp. *sakei* | SS78 | Sample 29 | Fermented vegetable | Radish kimchi |
| *L*. *sakei* subsp. *sakei* | SS79 | Sample 29 | Fermented vegetable | Radish kimchi |
| *L*. *sakei* subsp. *sakei* | SS80 | Sample 29 | Fermented vegetable | Radish kimchi |
| *L*. *sakei* subsp. *sakei* | SS81 | Sample 29 | Fermented vegetable | Radish kimchi |
| *L*. *sakei* subsp. *sakei* | SS76 | Sample 30 | Fermented vegetable | Young radish kimchi |
| *L*. *sakei* subsp. *sakei* | SS77 | Sample 30 | Fermented vegetable | Young radish kimchi |
| *L*. *sakei* subsp. *sakei* | SS82 | Sample 30 | Fermented vegetable | Young radish kimchi |
| *L*. *sakei* subsp. *sakei* | SS83 | Sample 30 | Fermented vegetable | Young radish kimchi |
| *L*. *sakei* subsp. *sakei* | SS87 | Sample 31 | Fermented vegetable | Radish kimchi |
| *L*. *sakei* subsp. *sakei* | SS88 | Sample 31 | Fermented vegetable | Radish kimchi |
| *L*. *sakei* subsp. *sakei* | SS89 | Sample 31 | Fermented vegetable | Radish kimchi |
| *L*. *sakei* subsp. *sakei* | SS90 | Sample 31 | Fermented vegetable | Radish kimchi |
